# Self-Harm Among School-Going Adolescent Survivors of Sexual Violence Victimisation: A Cross-Sectional Study

**DOI:** 10.3389/fsoc.2021.605865

**Published:** 2021-05-20

**Authors:** Emmanuel Nii-Boye Quarshie

**Affiliations:** ^1^Department of Psychology, University of Ghana, Accra, Ghana; ^2^School of Psychology, University of Leeds, Leeds, United Kingdom

**Keywords:** adolescents, Ghana, self-harm, sexual abuse, sexual violence, social adversity, suicide attempt, suicide

## Abstract

**Background:** A growing body of evidence from high-income contexts suggests a strong association between sexual violence victimisation and self-harm and eventual suicide. However, both sexual violence and self-harm among adolescents are still less researched in sub-Saharan African countries, including Ghana.

**Objectives:** To estimate the 12-month prevalence of self-harm, and to describe the associated factors and reported reasons for self-harm among school-going adolescent survivors of sexual violence victimisation during the previous 12 months in urban Ghana.

**Methods:** Analytic data came from a regional-based representative cross-sectional survey including in-school youth (*N* = 1,723) conducted in 2017 within the Greater Accra Region of Ghana. Of these, 297 (17.2%) self-reported sexual violence victimisation in the previous 12 months; this proportion of the participants (*n* = 297) was the focus of the current study. Items measuring sexual violence victimisation, self-harm, and correlates were adopted from the 2012 Ghana WHO–Global School-based Student Health Survey and the Child and Adolescent Self-harm in Europe Study. Data analysis involved multivariable logistic regression models.

**Results:** The estimate of self-harm ideation during the previous 12 months was 45.8% (95% CI: 40–52), whereas the estimate of self-harm behaviour was 38.7% (95% CI: 33–44). About two in five of the participants who reported self-harm wanted to die by their last episode of the behaviour. While bullying victimisation was associated with increased odds of self-harm ideation (aOR = 1.97, 95% CI 1.17, 3.31, *p* = 0.010) and behaviour (aOR = 2.76, 95% CI 1.59, 4.80, *p* < 0.001), weekly alcohol use (aOR = 2.56, 95% CI 1.32, 4.93, *p* = 0.005), conflict with parents (aOR = 2.30, 95% CI 1.28, 4.12, *p* = 0.005), and physical abuse victimisation (aOR = 1.80, 95% CI 1.03, 3.15, *p* = 0.037) showed strong associations with increased odds of self-harm behaviour in the past 12 months.

**Conclusions:** The evidence underscores the need for both universal and targeted multi-level intervention and prevention programmes to mitigate the offence of sexual violence and reduce the chances of self-harm among adolescent survivors of sexual violence in urban Ghana.

## Introduction

According to the World Health Organisation (WHO), sexual violence^1^ is “any sexual act, attempt to obtain a sexual act, unwanted sexual comments or advances, or acts to traffic, or otherwise directed, against a person's sexuality using coercion, by any person regardless of their relationship to the victim, in any setting, including but not limited to home and work” (Krug et al., 2002). The United Nations Children's Fund (UNICEF) estimates that across the world, about 13 million girls (representing one in every 20 girls) aged 15–19 years have experienced forced sex in their lifetime (UNICEF, 2014). As has been reported in high-income countries (UNICEF, 2014; UNICEF, UN Women, and Plan International, 2020), evidence from low- and middle-income countries (LAMICs), including those in sub-Saharan Africa suggests that boys are not exempt from sexual violence victimisation, even though girls are mostly abused (Veenema et al., 2015; Adjei and Saewyc, 2017). For example, in sub-Saharan Africa, self-reported lifetime prevalence estimates of sexual violence range between 33.5 and 36.2% among females and between 19.5 and 21.0% among males (Brown et al., 2009; Nguyen et al., 2019; Seff and Stark, 2019). In Ghana, a national household survey of sexual abuse victimisation has reported lifetime estimates of 38.2% among females and 19.4% among males, and 12-month estimates of 22.1% among females and 19.4% among males aged 15–19 years (Institute of Development Studies, 2016).

Besides poor academic outcomes and negative social and interpersonal problems, sexual violence victimisation among adolescents is associated with a wide-range of negative mental and physical health outcomes, including HIV and other sexually transmitted infections, broken bones, head trauma, depression, anxiety, and post-traumatic stress disorder (Wells et al., 1997; Daignault and Hebert, 2009; UNICEF, 2014; McLaughlin and Sheridan, 2016; Oram et al., 2017; McTavish et al., 2019; Nguyen et al., 2019; Seff and Stark, 2019). A growing body of evidence (mostly from high-income contexts) suggests that sexual violence victimisation is a strong risk for self-harm^2^, suicidal ideations, and eventual suicide among adolescents (Brown et al., 1999; Beautrais, 2000; Martin et al., 2004; Ystgaard et al., 2004; Klonsky and Moyer, 2008; Miller et al., 2013; Seff and Stark, 2019; Alix et al., 2020; Baiden et al., 2020). For example, evidence from Australia indicates that 54% of sexually abused (compared with 17% non-abused) adolescents report ever intentionally self-harming, while 32% of sexually abused adolescents versus 2% non-abused adolescents ever tried to take their own life five or more times (Martin et al., 2004).

A recent systematic review of the literature on self-harm, generally, among adolescents in sub-Saharan Africa reports a 12-month median prevalence estimate of 16.9% (interquartile range [IQR] = 11.5–25.5%) (Quarshie et al., 2020). However, thus far, only a few studies from countries in sub-Saharan Africa have reported self-harm specifically among adolescents with a history of sexual violence victimisation. An earlier study from Nigeria reported a 12-month prevalence estimate of suicidal ideation (40.8%) and suicidal attempt (24.2%) among in-school adolescents (Omigbodun et al., 2008). Recently, a cross-national survey has reported lifetime prevalence estimates of suicidal ideation and self-injury among adolescent victims of coerced or forced sexual initiation in three countries – Nigeria, Uganda, and Zimbabwe (Nguyen et al., 2019). Suicidal ideation ranged between 5.4 and 32% [Nigeria = 10.0% (5.4–14.7), Uganda = 23.8% (15.5–32.0), Zambia = 19.6% (11.9–27.3)], and self-injury ranged from 7.8 to 29.2% [Nigeria = 13.8% (7.8–19.8), Uganda = 13.3% (7.0–19.6), and Zambia = 20.9% (12.7–29.2)] (Nguyen et al., 2019).

Besides sexual violence victimisation being a single important risk factor for self-harm among adolescents, evidence from high-income countries suggests that sexually abused adolescents who are also exposed to negative school and peer-related factors (e.g., bullying), family dysfunctions (e.g., parent-child conflict), and mental health problems (e.g., anxiety, depression, alcohol, and substance use) are at an elevated risk of self-harm and even suicide (Yeo and Yeo, 1993; Boudewyn and Liem, 1995; Romans et al., 1995; Martin et al., 2004; Ystgaard et al., 2004). Compared to high-income countries, little is still known from African countries about other correlates in the form of adverse experiences that are associated with self-harm among sexually abused adolescents, while no published evidence exists from the region on self-reported reasons for self-harm among adolescent victims of sexual violence (Brown et al., 2009; Gage, 2013; Cluver et al., 2015; Nguyen et al., 2019; Seff and Stark, 2019; Quarshie et al., 2020). Taken together, given that the target to reduce suicide-related deaths has been prioritised in the sustainable development goals (UN Statistical Commission, 2016; Patel et al., 2018) and self-harm represents that single strongest risk for suicide (WHO, 2014; Naghavi and Global Burden of Disease Self-Harm Collaborators, 2019), it has become imperative to contribute sound and timely evidence through self-harm research, to inform intervention and prevention efforts, particularly, among young vulnerable groups including adolescent survivors of sexual violence in LAMICs, where 78% of the global suicide mortalities are reported from Bachmann (2018).

### Aims of Study

- Estimate the self-reported 12-month prevalence of self-harm ideation and behaviour among school-going adolescent^3^ survivors of sexual violence victimisation in urban Ghana.
- Identify the self-reported reasons/motivations for self-harm among school-going adolescent survivors of sexual violence victimisation.
- Describe some of the factors associated with self-harm among school-going adolescent survivors of sexual violence victimisation in urban Ghana.

## Materials and Methods

### Study Design and Participants

**Figure 1:**
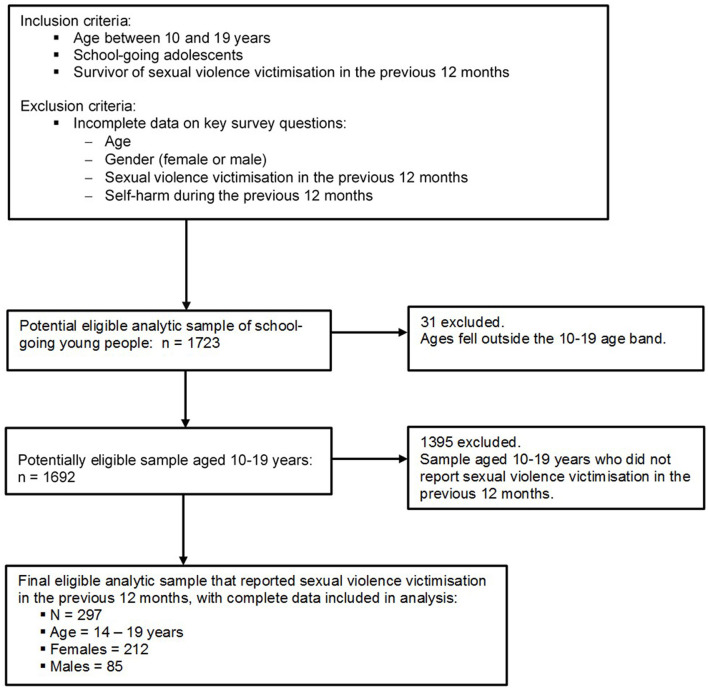
Flow diagram of analytic sample selection process and criteria.

This study draws data from a regionally representative large cross-sectional self-report school-based survey data collected in 2017 in the Greater Accra Region of Ghana—details of the methods and ethical issues have been reported elsewhere (Quarshie, 2019). In short, the design of the survey followed the community-agreed recommendations of Strengthening the Reporting of Observational Studies in Epidemiology—STROBE (Vandenbroucke et al., 2007). A two-stage cluster sampling approach was used. Firstly, 20 schools were selected, with probability proportional to their student population size. Secondly, classes were selected randomly also with probability proportional to class enrolment size, and all students in each selected class were invited to respond to the survey. Across the 20 schools, 1,723 students aged 13–21 years answered the questionnaire, although 1,928 students were invited to participate in the study—representing a response rate of 89.4%. Figure 1 shows the analytic sample selection procedure and the inclusion and exclusion criteria applied in the current study.

Of the 1,723 students, 1,692 fell within the adolescent age band criterion of 10–19 years. The survey assessed sexual violence victimisation with a binary-response (No or Yes) single-item: “Has anyone forced you (i.e., physically or verbally) to engage in sexual activities against your will during the past 12 months?” Two hundred and ninety-seven (297) of the 1,692 adolescent participants reported sexual violence victimisation during the 12 months preceding the survey—this represented the analytic sample for the current study (Figure 1).

### Measures

The questionnaire for the survey was in English, the language of instructions in schools in Ghana and the official language and lingua franca of the country. The measures included in this study covered demographic variables, lifestyle and social adversity factors, and outcome variables. The list of variables and specific questions used to assess them in the survey are provided in the Supplementary Table 1.

#### Exposures

##### Lifestyle Factors and Social Adversity

Nine binary (No or Yes) response rated lifestyle factors and variables assessing the experience of social and interpersonal adversities, adopted mainly from the 2012 WHO–Global School-based Student Health Survey (WHO-GSHS) questionnaire used in Ghana (WHO, 2015) were included. These included *weekly alcohol use* (In a typical week, how many times do you have at least one alcoholic drink?), *parental separation/divorce* (Have your parents separated or divorced during the past 12 months?), *conflict with parents* (Have you had any serious arguments or fights with either or both of your parents during the past 12 months?), *conflict between parents* (Have your parents had any serious arguments or fights during the past 12 months?), *bullying victimisation* (During the past 12 months, how many days were you bullied?), and *physical abuse victimisation* (Have you been seriously physically beaten during the past 12 months?)—see Supplementary Table 1.

##### Demographic Variables

Nine socio-demographic variables were included: gender (female or male), age, family structure (measured by father's number of wives), living arrangement, primary caretaker, caretaker's employment status, religious group, romantic relationship status, and sexual orientation—see Supplementary Table 1.

#### Outcomes

Self-harm during the previous 12 months was the main outcome variable in this study. This was assessed with a dichotomous (No = 0 or Yes = 1) response rated single item: “Have you, actually, intentionally harmed yourself (e.g., cutting, burning, or poisoning yourself, or tried to harm yourself in some other way, for example, hanging, jumping from height etc.) during the past 12 months?” This study also assessed the experience of self-harm ideation^4^ in the previous 12 months: “Have you thought about harming yourself (e.g., cutting, burning, or poisoning yourself, or considered harming yourself in some other way, for example, hanging, jumping from height etc.) during the past 12 months?” (No = 0 or Yes = 1). Given the lack of contextually developed measures of self-harm from LAMICs (Aggarwal and Berk, 2015; Aggarwal et al., 2017; Quarshie et al., 2020), the current study adopted the items measuring self-harm—behaviour and ideation—from the Child and Adolescent Self-harm in Europe studies (Hawton et al., 2002; Madge et al., 2008).

Similarly, a 15-item checklist of frequently reported reasons or motivations for self-harm were adopted from the WHO/EURO Multicentre Study on self-harm (Hjelmeland et al., 2002) and the Child and Adolescent Self-harm in Europe studies (Madge et al., 2008). A previous study from Africa has found the checklist to yield satisfactory consistency between Uganda and Norway (Hjelmeland et al., 2008). Some of the reasons on the checklist include “I wanted others to pay for the way they treated me,” “I wanted to die,” and “I wanted to persuade someone to change his/her mind” (see Supplementary Table 2). Drawing on the African cosmological worldview of aetiology—which attributes events to forces beyond the control of the individual—(Gyekye, 1995, 2003; Assimeng, 2010; Mbiti, 2015) the survey included an additional reason, “It was the work of the devil.”

### Data Collection Procedure

The questionnaire was expert-reviewed prior to the survey by a child-and-adolescent-health researcher, a developmental [child] psychologist, and a suicidologist in Ghana. In each participating school, the students were gathered in a larger classroom or assembly hall designated for the survey, with sitting arrangement spaced by reasonable distance to ensure participants had privacy while answering the questionnaire. On average, the completion of the questionnaire lasted between 22 and 45 min, after which each student placed their answered questionnaire in a box. The survey was conducted between May and September 2017.

### Ethics

The Ethics Committee for the Humanities, University of Ghana, Accra, Ghana approved this study (Ref. No: ECH078/16-17). In keeping with the research ethics of the Ghana Education Service, permissions were sought from the heads and management of all the participating schools. Prior to responding to the survey, each participant signed an actual written consent; parents and guardians of underage participants provided consent, while participants aged 17 years and younger assented prior to taking part in the study.

### Statistical Analysis

All statistical analyses were conducted using SPSS (version 26.0 for Windows). Missing responses were deleted list-wise during the analysis, as the loss of cases due to missing data was <5% (Graham, 2012). The Supplementary Table 1 shows the proportion of missing data for each variable included in the analysis. Frequencies and proportions were used to estimate the 12-month prevalence of self-harm (ideation and behaviour) and to assess the distribution of the socio-demographic variables, lifestyle and adversity factors, and reported reasons for self-harm. Similar to key evidence in self-harm research (Scoliers et al., 2009; Edmondson et al., 2016; Rasmussen et al., 2016; Taylor et al., 2018), the reported reasons for self-harm were grouped into “intrapersonal^5^” and “interpersonal^6^” (see Supplementary Table 2). Bivariable analysis using Chi square test was performed to assess the relationship between each of the exposure variables and self-harm (ideation and behaviour); Fisher's exact test was applied where the expected count of a cell was <5. Considering that age was a continuous variable, point-biserial correlation (r_pb_) test (Prematunga, 2012) was used to examine the possible bivariate relationship between age and self-harm (ideation and behaviour).

Adjusted and unadjusted multivariable logistic regression models were used to explore the possible associations between the exposures and the binary outcome variables, self-harm ideation and behaviour in the past 12 months. Variables included in the logistic regression models were considered regardless of the statistical significance of their bivariate relationships with the outcome variables (Sun et al., 1996; Babyak, 2004). The socio-demographic variables were included in the models as covariates. It must be noted though that in the initial models, “sexual orientation” was associated with very high or infinite odds ratios and confidence intervals, suggesting an existence of sparse data bias (Greenland et al., 2000, 2016a); thus, for a stable model, sexual orientation was excluded from the final logistic regression models. Results of the logistic regression were reported as odds ratios with 95% CIs (Greenland et al., 2016b) and *p* < 0.05 was used to determine statistically significant results.

## Results

### Characteristics of Participants

Table 1 presents the characteristics of the final analytic sample (*n* = 297); the participants were aged between 14 and 19 years (mean = 17.1; standard deviation = 1.1). There were more females (*n* = 212; 71.4%) than males (*n* = 85; 28.6%). Most participants (*n* = 177; 59.6%) reported having a father who had more than one wife.

**Table 1:** Distribution of sample across variables studied.

**Variable**	***n* (%)**
**Socio-demographic variables**	
Gender	
Male	85 (28.6)
Female	212 (71.4)
Family structure	
Father has 1 wife	177 (59.6)
Father has >1 wife	120 (40.4)
Living arrangement	
One or both parents	209 (70.4)
Other relative	63 (21.2)
Alone or with another person	25 (8.4)
Primary caretaker	
One or both parents	227 (76.4)
Other relative	42 (14.1)
Myself or another person	28 (9.4)
Primary caretaker's employment status	
Unemployed	29 (9.8)
Employed	268 (90.2)
Religious group	
Christian	278 (93.9)
Muslim	18 (6.1)
In romantic relationship	
No	120 (40.4)
Yes	177 (59.6)
Sexual orientation	
Heterosexual	278 (93.9)
Non-heterosexual	18 (6.1)
**Lifestyle and social adversity**	
Weekly alcohol use	
Never	230 (77.4)
≥1 drink	67 (22.6)
Parental separation/divorce	
No	157 (53.0)
Yes	139 (47.0)
Conflict with parents	
No	167 (56.2)
Yes	130 (43.8)
Conflict between parents	
No	130 (43.8)
Yes	167 (56.2)
Schoolwork problems	
No	137 (46.1)
Yes	160 (53.9)
Truancy	
≤5 days	255 (85.9)
>5 days	42 (14.1)
Breakup	
No	153 (51.7)
Yes	143 (48.3)
Bullying victimisation	
No	169 (56.9)
Yes	128 (43.1)
Physical abuse victimisation	
No	134 (45.1)
Yes	163 (54.9)

### Prevalence Estimates of Self-Harm and Bivariate Associations

The estimate of self-harm ideation during the previous 12 months was 45.8% (95% CI: 40–52), whereas the estimate of self-harm behaviour was 38.7% (95% CI: 33–44). As shown in Table 2, the estimates of self-harm ideation and behaviour were comparable between females and males; the differences did not reach statistical significance. Also, Table 2 shows that adolescent survivors of sexual violence victimisation who reported conflict with their parents were likely than those who reported no conflict with their parents to experience self-harm ideation [$\chi_{\left(1\right)}^{2}$ = 11.54, *p* = 0.001]. Bullying victimisation [$\chi_{\left(1\right)}^{2}$ = 9.91, *p* = 0.002], physical abuse victimisation [$\chi_{\left(1\right)}^{2}$ = 8.37, *p* = 0.004], and alcohol use [$\chi_{\left(1\right)}^{2}$ = 8.27, *p* = 0.004] showed significant bivariate relationships with self-harm ideation.

**Table 2:** Bivariable analysis (Chi square tests).

	**Self-harm ideation**				**Self-harm behaviour**			
	**No**	**Yes**	**χ^2^**	***p***	**No**	**Yes**	**χ^2^**	***p***
**Variable**	***n* (%)**	***n* (%)**			***n* (%)**	***n* (%)**		
**Socio-demographic variables**								
Gender			2.33	0.127			1.06	0.302
Male	52 (61.2)	33 (38.8)			56 (65.9)	29 (34.1)		
Female	109 (51.4)	103 (48.6)			126 (59.4)	86 (40.6)		
Family structure			1.44	0.231			1.81	0.179
Father has 1 wife	101 (57.1)	76 (42.9)			114 (64.4)	63 (35.6)		
Father has >1 wife	60 (50.0)	60 (50.0)			68 (56.7)	52 (43.3)		
Living arrangement			0.09	0.953			0.17	0.918
One or both parents	113 (54.1)	96 (45.9)			127 (60.8)	82 (39.2)		
Other relative	35 (55.6)	28 (44.4)			40 (63.5)	23 (36.5)		
Alone or with another person	13 (52.0)	12 (48.0)			15 (60.0)	10 (40.0)		
Primary caretaker			0.12	0.941			2.92	0.232
One or both parents	122 (53.7)	105 (46.3)			142 (62.6)	85 (37.4)		
Other relative	23 (54.8)	19 (45.2)			21 (50.0)	21 (50.0)		
Myself or another person	16 (57.1)	12 (42.9)			19 (67.9)	9 (32.1)		
Primary caretaker's employment status			0.08	0.777				
Unemployed	15 (51.7)	14 (48.3)			16 (55.2)	13 (44.8)	0.50	0.477
Employed	146 (54.5)	122 (45.5)			166 (61.9)	102 (38.1)		
Religious group			0.02	0.895			0.25	0.615
Christian	150 (54.0)	128 (46.0)			171 (61.5)	107 (38.5)		
Muslim	10 (55.6)	8 (44.4)			10 (55.6)	8 (44.4)		
In romantic relationship			0.00	0.990			1.76	0.185
No	65 (54.2)	55 (45.8)			79 (65.8)	41 (34.2)		
Yes	96 (54.2)	81 (45.8)			103 (58.2)	74 (41.8)		
Sexual orientation			3.31	0.069			**12.22**	**0.001**
Heterosexual	154 (55.4)	124 (44.6)			177 (63.7)	101 (36.3)		
Non-heterosexual	6 (33.3)	12 (66.7)			4 (22.2)	14 (77.8)		
**Lifestyle and social adversity factors**								
Weekly alcohol use			**8.27**	**0.004**			**13.85**	**0.000**
Never	135 (58.7)	95 (41.3)			154 (67.0)	76 (33.0)		
≥1 drink	26 (38.8)	41 (61.2)			28 (41.8)	39 (58.2)		
Parental separation/divorce			0.001	0.926			0.12	0.726
No	85 (54.1)	72 (45.9)			98 (62.4)	59 (37.6)		
Yes	76 (54.7)	63 (45.3)			84 (60.4)	55 (39.6)		
Conflict with parents			**11.54**	**0.001**			**20.08**	**0.000**
No	105 (62.9)	62 (37.1)			121 (72.5)	46 (27.5)		
Yes	56 (43.1)	74 (56.9)			61 (46.9)	69 (53.1)		
Conflict between parents			3.12	0.077			**5.02**	**0.025**
No	78 (60.0)	52 (40.0)			89 (68.5)	41 (31.5)		
Yes	83 (49.7)	84 (50.3)			93 (55.7)	74 (44.3)		
Schoolwork problems			**6.28**	**0.012**			**4.67**	**0.031**
No	85 (62.0)	52 (38.0)			93 (67.9)	44 (32.1)		
Yes	76 (47.5)	84 (52.5)			89 (55.6)	71 (44.4)		
Truancy			1.58	0.208			0.01	0.928
≤5 days	142 (55.7)	113 (44.3)			156 (61.2)	99 (38.8)		
>5 days	19 (45.2)	23 (54.8)			26 (61.9)	16 (38.1)		
Breakup			**4.71**	**0.030**			**4.06**	**0.044**
No	92 (60.1)	61 (39.9)			102 (66.7)	51 (33.3)		
Yes	68 (47.6)	75 (52.4)			79 (55.2)	64 (44.8)		
Bullying victimisation			**9.91**	**0.002**			**19.67**	**0.000**
No	105 (62.1)	64 (37.9)			122 (72.2)	47 (27.8)		
Yes	56 (43.8)	72 (56.3)			60 (46.9)	68 (53.1)		
Physical abuse victimisation			**8.37**	**0.004**			**11.05**	**0.001**
No	85 (63.4)	49 (36.6)			96 (71.6)	38 (28.4)		
Yes	76 (46.6)	87 (53.4)			86 (52.8)	77 (47.2)		

*χ^2^ = Chi square, boldface = statistically significant results*.

Similarly, adolescent survivors of sexual violence victimisation who reported conflict with their parents were likely than those who reported no conflict with their parents to report self-harm behaviour [$\chi_{\left(1\right)}^{2}$ = 20.08, *p* < 0.001]. Participants who experienced bullying victimisation [$\chi_{\left(1\right)}^{2}$ = 19.67, *p* < 0.001], reported alcohol use [$\chi_{\left(1\right)}^{2}$ = 13.85, *p* < 0.001], and physical abuse victimisation [$\chi_{\left(1\right)}^{2}$ = 11.05, *p* = 0.001], and those who identified as non-heterosexual [$\chi_{\left(1\right)}^{2}$ = 12.22, *p* = 0.001] were also likely to report self-harm behaviour during the previous 12 months. Age showed no statistically significant bivariate relationship with neither self-harm ideation (*r*_pb_ = −0.045, *N* = 297, *p* = 0.438) nor self-harm behaviour (*r*_pb_ = −0.14, *N* = 297, *p* = 0.808).

### Reported Reasons/Motivations for Last Episode of Self-Harm

The participants reported multiple reasons, including both intrapersonal and personal motives, for their last episode of self-harm—see Table 3.

**Table 3:** Reported reasons/motivations for last episode of self-harm behaviour.

		**Sample** (^*^n = 115)
**No**.	**Reported reason**	***n* (%)**
1	My thoughts were so unbearable, I could not endure them any longer	70 (60.9)
2	It seemed that I lost control of myself, and I do not know why I did it	38 (33.0)
3	The situation was so unbearable that I could not think of any other alternative	52 (45.2)
4	I wanted to get away for a while from an unacceptable situation	42 (36.5)
5	I wanted to sleep for a while	14 (12.2)
6	I wanted to punish myself	2 (1.7)
7	I wanted to die	47 (40.9)
8	I wanted to show someone how much I loved him/her	26 (22.6)
9	I wanted others to know how desperate I felt	26 (22.6)
10	I wanted to get help from someone	28 (24.3)
11	I wanted to know if someone really cared about me	58 (50.4)
12	I wanted others to pay for the way they treated me	33 (28.7)
13	I wanted to make someone feel guilty	30 (26.1)
14	I wanted to persuade someone to change his/her mind	24 (20.9)
15	I wanted to make things easier for others	28 (24.3)
16	It was the work of the devil	10 (8.7)
	**Reporting at least one type of reason**	
17	Intrapersonal	110 (95.6)
18	Interpersonal	97 (84.3)

* *Denominator (n) for computation of proportion is frequency of self-harm behaviour in the past 12-months*.

Generally, the participants checked more intrapersonal (95.6%) than interpersonal (84.3%) reasons for their self-harm behaviour. Specifically, the intrapersonal reason “My thoughts were so unbearable, I could not endure them any longer” was most frequently reported (60.9%), while the most frequently reported interpersonal reason was “I wanted to know if someone really cared about me” (50.4%). Notably, 40.9% of the 115 participants who reported self-harm behaviour indicated their motivation as “I wanted to die.” In other words, about two in five wanted to die by their self-harm.

### Associations of Self-Harm

#### Self-Harm Ideation

As shown in Table 4, no socio-demographic factor was observed to have a statistically significant association with neither increased nor reduced odds of self-harm ideation in both the unadjusted and adjusted models.

**Table 4:** Multivariate associations: self-harm ideation.

	**Model 1: Unadjusted**				**Model 2: Adjusted**			
	**β**	**OR**	**95% CI**	***p*-value**	**β**	**aOR**	**95% CI**	***p*-value**
**Socio-demographic variables**								
Gender	0.34	1.41	0.83, 2.38	0.200	0.37	1.44	0.81, 2.59	0.217
Age	−0.09	0.914	0.73, 1.14	0.434	−0.13	0.86	0.67, 1.10	0.239
Family structure	0.28	1.32	0.81, 2.16	0.258	0.19	1.21	0.68, 2.15	0.509
Living arrangement								
One or both parents	Reference			Reference				
Other relative	−0.06	0.94	0.47, 1.86	0.856	0.20	1.22	0.56, 2.66	0.613
Alone or with another person	0.199	1.22	0.47, 3.15	0.680	0.16	1.17	0.42, 3.23	0.760
Primary caretaker								
One or both parents	Reference			Reference				
Other relative	−0.07	0.93	0.42, 2.06	0.865	−0.08	0.92	0.39, 2.18	0.859
Myself or another person	−0.17	0.84	0.33, 2.12	0.716	−0.35	0.70	0.25, 1.93	0.490
Primary caretaker's employment status	−0.08	0.92	0.41, 2.04	0.835	−0.23	0.79	0.33, 1.94	0.614
Religious group	0.00	1.00	0.38, 2.65	0.999	−0.09	0.91	0.31, 2.63	0.860
In romantic relationship	0.04	1.04	0.63, 1.69	0.882	−0.16	0.85	0.48, 1.52	0.590
**Lifestyle and social adversity factors**								
Weekly alcohol use	0.51	1.67	0.91, 3.04	0.096	0.60	1.82	0.97, 3.42	0.061
Parental separation/divorce	−0.32	0.72	0.43, 1.21	0.217	−0.42	0.65	0.37, 1.16	0.148
Conflict with parents	**0.52**	**1.68**	**1.01, 2.81**	**0.047**	0.51	1.67	0.97, 2.89	0.064
Conflict between parents	0.27	1.32	0.78, 2.21	0.296	0.21	1.23	0.71, 2.14	0.453
Schoolwork problems	0.39	1.48	0.90, 2.44	0.122	0.34	1.40	0.84, 2.36	0.200
Truancy	0.08	1.08	0.52, 2.24	0.827	0.12	1.13	0.53, 2.38	0.752
Breakup	0.39	1.49	0.89, 2.49	0.127	0.49	1.63	0.91, 2.91	0.097
Bullying victimisation	**0.62**	**1.86**	**1.13, 3.07**	**0.014**	**0.68**	**1.97**	**1.17, 3.31**	**0.010**
Physical abuse victimisation	0.44	1.55	0.94, 2.56	0.085	0.45	1.57	0.94, 2.64	0.085

*β, beta value; Aor, adjusted odds ratio; CI, Confidence interval; boldface = statistically significant results*.

However, conflict with parents was associated with increased odds of self-harm ideation (OR = 1.68, 95% CI 1.01, 2.81, *p* = 0.047), while bullying victimisation was associated with increased odds of self-harm ideation in both the unadjusted (OR = 1.86, 95% CI 1.13, 3.07, *p* = 0.014) and adjusted (aOR = 1.97, 95% CI 1.17, 3.31, *p* = 0.010) models.

#### Self-Harm Behaviour

In the unadjusted model, bullying victimisation (OR = 2.60, 95% CI 1.54, 4.39, *p* < 0.001), weekly alcohol use (OR = 2.34, 95% CI 1.25, 4.36, *p* = 0.008), conflict with parents (OR = 2.27, 95% CI 1.32, 3.89, *p* = 0.003), and physical abuse victimisation (OR = 1.71, 95% CI 1.00, 2.92, *p* = 0.048) showed strong associations with increased odds of self-harm behaviour in the past 12 months—see Table 5.

**Table 5:** Multivariate associations: self-harm behaviour.

	**Model 1: Unadjusted**				**Model 2: Adjusted**			
	**β**	**OR**	**95% CI**	***p*-value**	**β**	**aOR**	**95% CI**	***p*-value**
**Socio-demographic variables**								
Gender	0.15	1.16	0.67, 1.99	0.597	0.12	1.13	0.60, 2.12	0.700
Age	−0.07	0.93	0.73, 1.17	0.534	−0.13	0.88	0.67, 1.14	0.341
Family structure	0.29	1.34	0.81, 2.21	0.255	0.24	1.27	0.69, 2.36	0.440
Living arrangement								
One or both parents	Reference			Reference				
Other relative	−0.61	0.54	0.26, 1.14	0.106	−0.44	0.64	0.26, 1.58	0.336
Alone or with another person	0.24	1.27	0.48, 3.39	0.625	0.24	1.27	0.43, 3.75	0.663
Primary caretaker								
One or both parents	Reference			Reference				
Other relative	0.75	2.12	0.93, 4.83	0.072	0.90	2.47	0.98, 6.21	0.055
Myself or another person	−0.41	0.66	0.25, 1.79	0.418	−0.67	0.51	0.16, 1.59	0.247
Primary caretaker's employment status	−0.29	0.74	0.33, 1.68	0.480	−0.57	0.56	0.21, 1.49	0.249
Religious group	0.34	1.41	0.52, 3.80	0.495	0.30	1.35	0.44, 4.14	0.598
In romantic relationship	0.38	1.46	0.87, 2.44	0.147	0.37	1.45	0.77, 2.73	0.245
**Lifestyle and social adversity factors**								
Weekly alcohol use	**0.85**	**2.34**	**1.25, 4.36**	**0.008**	**0.94**	**2.56**	**1.32, 4.93**	**0.005**
Parental separation/divorce	−0.27	0.76	0.44, 1.31	0.326	−0.42	0.65	0.35, 1.21	0.176
Conflict with parents	**0.82**	**2.27**	**1.32, 3.89**	**0.003**	**0.83**	**2.30**	**1.28, 4.12**	**0.005**
Conflict between parents	0.29	1.33	0.77, 2.32	0.307	0.20	1.22	0.67, 2.21	0.508
Schoolwork problems	0.31	1.36	0.80, 2.32	0.256	0.33	1.39	0.79, 2.46	0.244
Truancy	−0.60	0.54	0.25, 1.20	0.134	−0.73	0.48	0.21, 1.11	0.088
Breakup	0.40	1.49	0.86, 2.57	0.150	0.37	1.45	0.77, 2.70	0.244
Bullying victimisation	**0.96**	**2.60**	**1.54, 4.39**	**0.000**	**1.02**	**2.76**	**1.59, 4.80**	**0.000**
Physical abuse victimisation	**0.54**	**1.71**	**1.00, 2.92**	**0.048**	**0.59**	**1.80**	**1.03, 3.15**	**0.037**

*β, beta value; aOR, adjusted odds ratio; CI, Confidence interval; boldface = statistically significant results*.

Similarly, in the adjusted model, bullying victimisation (aOR = 2.76, 95% CI 1.59, 4.80, *p* < 0.001), weekly alcohol use (aOR = 2.56, 95% CI 1.32, 4.93, *p* = 0.005), conflict with parents (aOR = 2.30, 95% CI 1.28, 4.12, *p* = 0.005), and physical abuse victimisation (aOR = 1.80, 95% CI 1.03, 3.15, *p* = 0.037) showed strong associations with increased odds of self-harm behaviour in the past 12 months. However, in both unadjusted and adjusted models, no socio-demographic variable was observed to have a statistically significant association with self-harm behaviour.

Notably, bullying victimisation emerged as the only factor with a strong association with both self-harm ideation and behaviour across both unadjusted and adjusted models (Tables 4, 5).

## Discussion

Taken together, the findings of this study show that: (1) about four out of 10 school-going adolescent survivors of sexual violence victimisation reported self-harm ideation, while three out of 10 reported self-harm behaviour in the previous 12 months; (2) school-going adolescent survivors of sexual violence victimisation reported more intrapersonal reasons for their last episode of self-harm, about two in five wanted to die by their self-harm; and (3) lifestyle and social adversity factors were associated with increased odds of self-harm ideation and behaviour; in particular, bullying victimisation showed a strong association with both self-harm ideation and behaviour.

The 12-month prevalence estimate of self-harm among adolescent survivors of sexual violence in the present study (38.7% [95% CI: 33–44%]) is higher, relative to the estimates of self-harm among the general population of adolescents across the region of sub-Saharan Africa [median: 16.9%; IQR = 11.5–25.5%] (Quarshie et al., 2020), and LAMICs [11.5–33.8%] (Aggarwal et al., 2017; Mannekote Thippaiah et al., 2020); it is still higher than the global estimates among the general population of adolescents [10.1–19.5%] (Lim et al., 2019). However, the estimate of self-harm in the present study is comparable to what has been reported among adolescent survivors of sexual violence in high-income countries; for example, Australia, where 54% intentionally self-harmed and 24% attempted suicide (Martin et al., 2004). Possibly, this evidence of higher prevalence estimates could be underscoring the importance of sexual violence victimisation as a critical risk for self-harm among adolescents (Klonsky and Moyer, 2008; Brown et al., 2009; Miller et al., 2013; UNICEF, 2014). The high prevalence estimate could also be supportive of the evidence that adolescent survivors of sexual violence experience significant emotional problems and psychological disturbance, which in turn lead up to self-harm (Brown et al., 1999; Beautrais, 2000).

In the light of both the African and global literature, the current study represents the first effort at reporting evidence on the motivations of school-going adolescent survivors of sexual violence for engaging in self-harm. Many of the participants reported constriction of thought and inability to generate alternative courses of action, but also experienced the motivation to die. About two in five wanted to die by their self-harm. This finding is comparable to evidence from high-income countries, where many adolescent survivors have reported death-intended self-harm behaviours (Brown et al., 1999; Beautrais, 2000; Martin et al., 2004; Ystgaard et al., 2004; Klonsky and Moyer, 2008; Miller et al., 2013; Seff and Stark, 2019; Alix et al., 2020; Baiden et al., 2020). For example, evidence from Australia indicates that 32% of sexually abused adolescents, compared to 2% non-abused adolescents, ever tried to take their own life five or more times (Martin et al., 2004).

Furthermore, this evidence supports the findings of earlier studies that, apart from clinically symptomatic depression and other internalising symptoms, the offence of sexual violence generates suicidal tendencies in adolescent survivors, as the abuse gravely affects the self-esteem and global self-worth of survivors (Serafini et al., 2015; McLaughlin and Sheridan, 2016; Mutavi et al., 2018a,b). Additionally, the finding that many of the adolescent survivors of sexual violence victimisation reported interpersonal reasons for their self-harm is to be expected, as many also had the motive of wanting “others to pay for the way they treated me.” This seeming vengeful motive could be indicative of the negative emotional responses and anger that are often experienced by survivors of sexual violence victimisation (Coyle et al., 2014; Boyle and Clay-Warner, 2018). However, further studies (involving qualitative approaches) are needed to explore the meanings of the self-harm motive “I wanted to make things easier for others” as reported by the adolescent survivors of sexual violence in the current study. While further research is needed to expand our understanding of this finding, the evidence is critical for the design and planning of intervention and prevention programmes against the onset of self-harm ideation and transition to self-harm behaviour among this vulnerable population of young people.

Regarding factors associated with self-harm among school-going adolescent survivors of sexual violence, this study has shown that lifestyle factors (such as weekly alcohol use) and adverse social experiences (i.e., bullying victimisation, conflict with parents, and physical abuse victimisation) have strong associations with increased odds of self-harm. This evidence is consistent with findings from a longitudinal study from South Africa (Cluver et al., 2015) and a global systematic review (Serafini et al., 2015) that multiple or cumulative exposures to adverse experiences among adolescents is associated with an elevated risk of self-harm. The *dimension of adversity theory* posits that exposure to multiple adverse experiences could fall between *threat* and *deprivation*, both of which could potentially compromise the (later) emotional well-being, cognitive development, and mental health of the individual (McLaughlin and Sheridan, 2016). Threat experiences (e.g., weekly alcohol use, sexual abuse victimisation, conflict with parents, bullying victimisation, and physical abuse victimisation) involve harm or threat of harm, while deprivation experiences involve absence of expected input from the environment (e.g., neglect, poverty). Each of these experiences of threat and deprivation are varied in severity and frequency, but they all involve (threat of) harm (McLaughlin and Sheridan, 2016; Lambert et al., 2017). In the current study, possibly, weekly alcohol use, conflict with parents, and physical abuse victimisation were threats that simultaneously compromised the resilience and resistance of the adolescents, thereby leading up to self-harm.

The finding that bullying victimisation was uniquely associated with both self-harm ideation and behaviour is worth some comments. This finding is worrying but not totally surprising, considering that sexual abuse is stigmatised and connotes unpleasant social labels and victim-blaming (UNICEF, 2014; Williamson and Serna, 2018). Evidence from Ghana indicates that entrenched patriarchal beliefs and rape myth acceptance tend to allow victim-blaming to fester (Boakye, 2009a,b). Thus, compared to non-abused adolescents, adolescent survivors of sexual violence victimisation tend to be at increased vulnerability to bullying and its attendant negative mental health outcomes, including self-blame, self-dislike, and potentially self-destructive behaviours (Hébert et al., 2016).

Relatedly, conflict between parents and their adolescent survivors of sexual violence victimisation is also of concern but not totally surprising, as there is evidence to suggest that some parents are less supportive of their child who has been sexually abused (Elliott and Carnes, 2001; Ullman, 2002). Recent evidence from Ghana suggests that some families of sexually abused children tend to misconstrue the abuse as a consequence of failed parenting and as a betrayal by their abused child; this often leads up to conflict between some parents and their sexually abused child (Tetteh and Markwei, 2018). The emergence of parent-child conflict in the context of child sexual abuse could lead to self-blame and other self-directed negative emotions and impulses on the part of the abused child, which in turn may lead up to self-harm.

Certainly, further expansive research is needed to extend the clarification of the evidence on the association between sexual violence victimisation and self-harm among adolescents in Ghana, but also across other countries in sub-Saharan Africa. Put together, the evidence of the current study highlights the need for universal and targeted multi-level intervention and prevention programmes at the individual level (e.g., teaching adolescents self-defence for reduced risk of sexual violence and bullying victimisation, helping adolescents to avoid alcohol and substance use, and teaching them emotional and social skills), family context (e.g., teaching effective parent-child communication characterised by emotional disclosure, and teaching supportive parenting skills), and the school environment (e.g., training school staff on promoting pro-mental health school climate, and enforcing existing child protection policies to stop bullying, sexual violence, and physical abuse of students). At the macro-level, it is strongly recommended that the government of Ghana and its stakeholder agencies (e.g., Ghana Education Service, Department of Social Welfare, Ministry of Gender, Children and Social Protection) continue to give full cooperation to the support lend by UNICEF toward the effective implementation of various child protection policies and social intervention programmes (National Development Planning Commission, UNICEF, and Ghana Statistical Service, 2020; UNICEF, 2020a,b).

Broadly, this study is in response to the recommendation for further evidence on self-harm among sexually abused young people, particularly, in LAMICs (Klonsky and Moyer, 2008; Miller et al., 2013; UNICEF, 2014; WHO, 2014; Nguyen et al., 2019). This study is the first from Ghana to report estimates of self-harm among adolescent survivors of sexual violence, and the first to contribute evidence to the global literature on reported reasons or self-reported motives for self-harm by adolescent survivors of sexual violence. Nonetheless, the interpretation and adoption of the evidence presented in this study must be done cautiously, owing to some notable limitations.

There is evidence from Ghana to suggest that the general non-supportive social environment precludes survivors of sexual violence from disclosing their abuse (Boakye, 2009b), and the criminalised and highly stigmatised status of self-harm (attempted suicide) also leads to increased non-disclosure and socially desirable responses among research participants (Osafo et al., 2015). The implication could be an underestimation of both self-reported sexual violence victimisation and self-harm. Notably though, the position of the current study is that, plausible non-reporting and underestimation might be low, as participants in the current study were allowed reasonable privacy in responding to the survey: a self-report anonymous questionnaire was used, teachers were not allowed into the location of the survey, and participants sat far apart from one another during the survey.

Furthermore, in this cross-sectional survey both the outcome and exposure variables were measured at the same point in time; this means that the findings reported in this study cannot support causal interpretations. It is also important to point out that the current study failed to include some key variables related to mental health—for example, depression, hopelessness, and anxiety—which have been found to mediate the association between sexual violence victimisation and self-harm (Martin et al., 2004; Klonsky and Moyer, 2008; Miller et al., 2013; Serafini et al., 2017). Whereas, the evidence of this study may be generalisable across urban in-school adolescents, they may not necessarily be applicable to adolescents in rural Ghana. Also, it is plausible that the single-item measure used in this study to assess sexual violence victimisation might have been partly inadequate in capturing the full nuances of sexual violence victimisation; similarly, even though single-item measures of self-harm facilitate the screening of larger research participants at a time, potentially, such measure could lead to misclassification of self-harm behaviours (Millner et al., 2015; Hom et al., 2016). Future, studies may consider applying validated checklist or multi-item measures with satisfactory ecological validity and relevance. Lastly, of the 297 adolescent survivors of sexual violence included in this study, 71.4% (*n* = 212) were females. While this disproportionate distribution potentially skews the results to be more applicable to females, it also precluded meaningful stratification and sub-group analysis of the data in terms of gender. Notably however, this observation is not intended to mean that the effects of sexual violence victimisation on females is more harmful than on males; sexual violence victimisation is troubling for both females and males in the immediate and long-terms.

Relatively, the estimates of self-harm among school-going adolescent survivors of sexual violence victimisation are higher; the motivations for self-harm are intrapersonal, including the motive to die. Social adversities in the family and school contexts present as strong factors associated with self-harm among school-going adolescent survivors of sexual violence victimisation. The evidence underscores the need for both universal and targeted multi-level intervention and prevention programmes to mitigate the offence of sexual violence and reduce the chances of self-harm among adolescent survivors of sexual violence in urban Ghana.

## Data Availability Statement

The raw data supporting the conclusions of this article will be made available by the authors, without undue reservation.

## Ethics Statement

The studies involving human participants were reviewed and approved by The Ethics Committee for the Humanities, University of Ghana, Accra, Ghana approved this study (Ref. No: ECH078/16-17). Written informed consent to participate in this study was provided by the participants' legal guardian/next of kin.

## Author Contributions

The author confirms being the sole contributor of this work and has approved it for publication.

## Conflict of Interest

The author declares that the research was conducted in the absence of any commercial or financial relationships that could be construed as a potential conflict of interest.

## Footnotes

^1^In the current study, “sexual violence”, “sexual abuse”, and “coerced sex” have been used interchangeably to mean the same phenomenon.

^2^Self-harm is used in the current study to mean any intentional “act of self-poisoning or self-injury carried out by an individual irrespective of motivation” [National Institute for Health and Care Excellence (NICE), 2012, p.14].

^3^This study applies WHO's chronological definition of an adolescent: an individual aged between 10 and 19 years (WHO, 2009).

^4^Self-harm ideation is taken to mean having thoughts to engage in self-harm (self-harm behaviour).

^5^Intrapersonal reasons for self-harm are related to attaining emotional or cognitive relief or punish self, etc.

^6^Interpersonal reasons for self-harm are related to seeking help, communicating distress, punishing others, or influencing the behaviour of others.
