# Supplementary material for: Self-Harm Among School-Going Adolescent Survivors of Sexual Violence Victimisation: A Cross-Sectional Study
Source: Front Sociol. 2021 May 20;6:605865. doi: 10.3389/fsoc.2021.605865 (PMC8174787; doi:10.3389/fsoc.2021.605865)
Supplement: Supplementary file 1 [file Data_Sheet_1.PDF]

e-Table 1. Coding of variables and missing data

Variables	Survey question	Response coding	Observation / Missing (%)
**Socio-demographic variables**			
Gender	What is your gender?	Male = 0; Female = 1	N = 297 / 0 (0)
Age	What is your age?	Coded continuously (13 – 21 years)	N = 297 / 0 (0)
Family Structure	How will you describe your family structure?	My father has 1 wife = 0<br>My father has > 1 wife = 1	N = 297 / 0 (0)
Living arrangement	What is your living arrangement? I live…	with one or both parents = 0<br>with other relative = 1<br>alone or with another person = 2	N = 297 / 0 (0)
Primary caretaker	Who is most responsible for taking care of your needs?	One or both parents = 0<br>Other relative = 1<br>Myself or with another person = 2	N = 297 / 0 (0)
Primary caretaker's employment status	What is the employment status of parent(s) or guardian?	Unemployed = 0; Employed = 1	N = 297 / 0 (0)
Religious group	What is your religious group?	Christian = 0; Muslim = 1	N = 296 / 1 (0.3)
In romantic relationship	Do you have a boyfriend or girlfriend?	No = 0; Yes = 1	N = 297 / 0 (0)
Sexual orientation	How would you describe your sexual orientation?	Heterosexual = 0<br>Non-heterosexual = 1	N = 296 / 1 (0.3)
**Lifestyle and social adversity**			
Weekly alcoholic drinks	In a typical week, how many times do you at least one alcoholic drink? (This includes drinking beer, akpeteshie, palm wine, pito, brukutu, gin, brandy, bonsamnsuo, yebudidi, schnapps, vodka, black label, bailey, alomo, club, ogidigidi, Guiness, Smirnoff, Hennessy, or Star. Drinking alcohol does not include drinking a few sips of wine for religious purposes. One "drink" is a glass of wine, a bottle of beer, a small glass of liquor, or a mixed drink).	Never drink = 0<br>1 or more drinks = 1	N = 297 / 0 (0)
Parental separation/divorce	Have your parents separated or divorced during the past 12 months?	No = 0; Yes = 1	N = 296 / 1 (0.3)
Conflict with parents	Have you had any serious arguments or fights with either or both of your parents during the past 12 months?	No = 0; Yes = 1	N = 297 / 0 (0)

e-Table 1. (continued)

Variables	Survey question	Response coding	Observation / Missing (%)
Conflict between parents	Have your parents had any serious arguments or fights during the past 12 months?	No = 0; Yes = 1	N = 297 / 0 (0)
School work problems	Have you had problems keeping up with schoolwork during the past 12 months?	No = 0; Yes = 1	N = 297 / 0 (0)
Truancy	During the past 12 months, on how many days were you absent from school without permission?	0-5 days = 0; > 5 days = 1	N = 297 / 0 (0)
Breakup	Have you had a break-up with a boyfriend or girlfriend during the past 12 months?	No = 0; Yes = 1	N = 296 / 1 (0.3)
Bullying victimisation	Have you been bullied at school or in your area during the past 12 months? Please note: Bullying occurs when a student or a group of students tease, threaten, spread rumours about, hit, shove, or hurt another student over and over again. It is not bullying when two students of about the same strength or power argue or fight or tease each other in a friendly and fun way.	No = 0; Yes = 1	N = 297 / 0 (0)
Physical abuse victimisation	Have you been seriously physically beaten during the past 12 months?	No = 0; Yes = 1	N = 297 / 0 (0)
**Outcome variables**			N = 297 / 0 (0)
Self-harm ideation in the previous 12 months	Have you thought about harming yourself (e.g., cutting, burning, or poisoning yourself, or considered harming yourself in some other way, for example, hanging, jumping from height etc.) during the past 12 months?	No = 0; Yes = 1	N = 297 / 0 (0)
Self-harm behaviour in the previous 12 months	Have you, actually, intentionally harmed yourself (e.g., cutting, burning, or poisoning yourself, or tried to harm yourself in some other way, for example, hanging, jumping from height etc.) during the past 12 months?	No = 0; Yes = 1	N = 297 / 0 (0)

e-Table 2. Categorisation of reasons/motivations for last episode of self-harm

Specific reasons/motivations	Grouped as
▪ My thoughts were so unbearable, I could not endure them any longer<br>▪ It seemed that I lost control of myself, and I do not know why I did it<br>▪ The situation was so unbearable that I could not think of any other alternative<br>▪ I wanted to get away for a while from an unacceptable situation<br>▪ I wanted to sleep for a while<br>▪ I wanted to punish myself<br>▪ I wanted to die	Intrapersonal reasons
▪ I wanted to show someone how much I loved him/her<br>▪ I wanted others to know how desperate I felt<br>▪ I wanted to get help from someone<br>▪ I wanted to know if someone really cared about me<br>▪ I wanted others to pay for the way they treated me<br>▪ I wanted to make someone feel guilty<br>▪ I wanted to persuade someone to change his/her mind<br>▪ I wanted to make things easier for others	Interpersonal reasons
